# Supplementary material for: Impact of HLA Polymorphism on the Immune Response to Bacillus Anthracis Protective Antigen in Vaccination versus Natural Infection
Source: Vaccines (Basel). 2022 Sep 20;10(10):1571. doi: 10.3390/vaccines10101571 (PMC9610610; doi:10.3390/vaccines10101571)
Supplement: Supplementary file 1 [file vaccines-10-01571-s001.zip › vaccines-1858276-supplementary.pdf]

vaccines

MDPI

**Supplementary Materials:** The following are available online at www.mdpi.com/xxx/s1,

**Table S1: Study inclusion and exclusion criteria**

| | |
|---|---|
| Inclusion criteria | |
| 1. | Male or female aged 18-55 years inclusive. |
| 2. | Able to understand and give informed consent to participate. |
| 3. | Participants with either; a history of recovery following hospitalisation with confirmed, culture-positive, cutaneous *B. anthracis* infection, or a history of vaccination in accordance with the UK schedule for the Anthrax Vaccine Precipitated (AVP) vaccine. |
| Exclusion criteria | |
| 1. | Subjects with known or suspected immunodeficiency. |
| 2. | Presence of any clinically significant medical condition or prescribed drug deemed by the study doctor to make the participant unsuitable for the study. |
| 3. | History or evidence of drug abuse, including a positive urine drug screen (for Cocaine, Amphetamines, Benzodiazepines, Methamphetamines, Tricyclics, Methadone, Barbiturates, Cannabis, Morphine). |
| 4. | Positive test for human immunodeficiency virus (HIV), and/or hepatitis B and/or hepatitis C. |
| 5. | Donation of blood or blood products for a period of 4 weeks prior to participation in the study. History of receiving blood or plasma transfusions, or pooled gamma-globulin in the previous 3 months, or a need for blood or plasma transfusions during the study. |

vaccines

MDPI

**Table S2. CD4+ T cell responses to *B. anthracis* PA epitopes in AVP vaccinees**

| Human cohorts | HLA class II | | | | | | T cell response to anthrax PA domain I-IV epitopes, SFC/$10^6$ cells | | | | | | | | | | | | | | | | | | | | | | | | | |
|---|---|---|---|---|---|---|---|---|---|---|---|---|---|---|---|---|---|---|---|---|---|---|---|---|---|---|---|---|---|---|---|---|
| | HLA-DRB1* | | HLA-DRB3*/4*/5* | | HLA-DQB1* | | 11-30 | 21-40 | 41-60 | 61-80 | 81-100 | 141-160 | 161-180 | 191-210 | 221-240 | 241-260 | 261-280 | 301-320 | 321-340 | 361-380 | 391-410 | 421-440 | 431-450 | 491-510 | 501-520 | 521-540 | 561-580 | 601-620 | 621-640 | 631-650 | 641-660 | 671-690 |
| AVP vaccinee 1 | 11 | 15 | 51 | 52 | 6 | 7 | 0 | 0 | 0 | 0 | 0 | 0 | 219 | 266 | 0 | 0 | 0 | 0 | 0 | 190 | 217 | 0 | 284 | 0 | 0 | 0 | 0 | 0 | 0 | 242 | 242 | 313 |
| AVP vaccinee 2 | 11 | 15 | 51 | 52 | 6 | 7 | 0 | 891 | 0 | 0 | 0 | 0 | 0 | 0 | 0 | 0 | 0 | 0 | 0 | 0 | 0 | 0 | 0 | 0 | 819 | 0 | 0 | 0 | 0 | 0 | 0 | 0 |
| AVP vaccinee 3 | 11 | 13 | 52 | - | 6 | 7 | 1247 | 1177 | 1057 | 977 | 895 | 933 | 0 | 0 | 1159 | 1077 | 485 | 681 | 169 | 521 | 0 | 1123 | 1199 | 1109 | 1133 | 857 | 821 | 1015 | 1077 | 0 | 0 | 1079 |
| AVP vaccinee 4 | 15 | 7 | 51 | 53 | 2 | 6 | 0 | 0 | 0 | 0 | 0 | 0 | 0 | 0 | 0 | 519 | 0 | 309 | 0 | 0 | 0 | 0 | 0 | 0 | 0 | 0 | 0 | 0 | 0 | 0 | 0 | 0 |
| AVP vaccinee 5 | 103 | 17 | 52 | - | 2 | 5 | 0 | 0 | 0 | 0 | 0 | 0 | 0 | 0 | 0 | 0 | 0 | 0 | 0 | 0 | 0 | 0 | 0 | 0 | 0 | 0 | 0 | 0 | 0 | 0 | 0 | 0 |
| AVP vaccinee 6 | 1 | 13 | 52 | - | 5 | 6 | 0 | 0 | 0 | 0 | 0 | 0 | 0 | 0 | 0 | 0 | 0 | 0 | 0 | 0 | 0 | 0 | 0 | 0 | 0 | 0 | 0 | 0 | 0 | 0 | 0 | 0 |
| AVP vaccinee 7 | 11 | 15 | 51 | 52 | 6 | 7 | 0 | 0 | 0 | 0 | 0 | 0 | 0 | 0 | 0 | 0 | 0 | 0 | 0 | 0 | 0 | 0 | 0 | 0 | 0 | 0 | 0 | 0 | 0 | 0 | 0 | 0 |
| AVP vaccinee 8 | 1 | - | - | - | 5 | - | 0 | 0 | 0 | 0 | 0 | 0 | 0 | 0 | 0 | 0 | 0 | 0 | 0 | 0 | 0 | 0 | 0 | 0 | 0 | 0 | 0 | 0 | 0 | 0 | 0 | 0 |
| AVP vaccinee 9 | 4 | 12 | 52 | 53 | 7 | 8 | 0 | 0 | 0 | 0 | 0 | 0 | 0 | 0 | 0 | 0 | 0 | 0 | 0 | 0 | 0 | 0 | 0 | 0 | 0 | 0 | 0 | 0 | 0 | 0 | 0 | 0 |
| AVP vaccinee 10 | 7 | 15 | 51 | 53 | 2 | 6 | 0 | 0 | 0 | 0 | 0 | 0 | 0 | 0 | 0 | 0 | 0 | 0 | 0 | 0 | 0 | 0 | 0 | 0 | 0 | 0 | 0 | 0 | 0 | 0 | 0 | 0 |

**Table S3. CD4+ T cell responses to *B. anthracis* PA epitopes in anthrax-recovered patients**

| Human cohorts | HLA class II | | | | | | T cell response to anthrax PA domain I-IV epitopes, SFC/$10^6$ cells | | | | | | | | | | | | | | |
|---|---|---|---|---|---|---|---|---|---|---|---|---|---|---|---|---|---|---|---|---|---|
| | HLA-DRB1* | | HLA-DRB3*/4*/5* | | HLA-DQB1* | | 1-20 | 11-30 | 21-40 | 31-50 | 41-60 | 51-70 | 61-80 | 71-90 | 81-100 | 91-110 | 101-120 | 121-140 | 131-150 | 141-160 | 151-170 |
| Infected donor 1 | 11 | 13 | 52 | - | 6 | 7 | 0 | 0 | 0 | 0 | 0 | 0 | 0 | 0 | 0 | 0 | 0 | 0 | 0 | 0 | 0 |
| Infected donor 2 | 4 | - | 53 | - | 8 | - | 299 | 330 | 301 | 263 | 224 | 0 | 203 | 0 | 0 | 0 | 0 | 0 | 304 | 299 | 417 |
| Infected donor 3 | 4 | 14 | 52 | 53 | 5 | 8 | 0 | 0 | 0 | 0 | 0 | 0 | 0 | 486 | 0 | 454 | 0 | 0 | 0 | 0 | 0 |
| Infected donor 4 | 15 | - | 51 | - | 6 | - | 0 | 0 | 0 | 0 | 0 | 0 | 0 | 0 | 0 | 0 | 0 | 0 | 0 | 0 | 0 |
| Infected donor 5 | 8 | 11 | 52 | - | 7 | - | 0 | 0 | 0 | 0 | 0 | 0 | 0 | 0 | 0 | 0 | 0 | 0 | 0 | 0 | 977 |
| Infected donor 6 | 11 | 13 | 52 | - | 6 | 7 | 0 | 0 | 0 | 0 | 0 | 0 | 0 | 0 | 0 | 0 | 0 | 581 | 0 | 0 | 0 |
| Infected donor 7 | 4 | 14 | 52 | 53 | 5 | - | 0 | 0 | 1017 | 0 | 971 | 723 | 1193 | 957 | 1225 | 949 | 793 | 451 | 0 | 505 | 691 |
| Infected donor 8 | 1 | 16 | 51 | - | 5 | - | 0 | 0 | 0 | 0 | 759 | 784 | 0 | 0 | 0 | 0 | 0 | 0 | 0 | 0 | 0 |
| Infected donor 9 | 15 | 13 | 51 | 52 | 6 | - | 0 | 0 | 0 | 0 | 0 | 0 | 0 | 0 | 0 | 0 | 0 | 0 | 0 | 0 | 0 |

| Human cohorts | T cell response to anthrax PA domain I-IV epitopes, SFC/$10^6$ cells | | | | | | | | | | | | | | | | |
|---|---|---|---|---|---|---|---|---|---|---|---|---|---|---|---|---|---|
| | 148-167 | 168-187 | 161-180 | 171-190 | 181-200 | 191-210 | 211-230 | 221-240 | 231-250 | 241-260 | 261-280 | 271-290 | 281-300 | 301-320 | 311-330 | 321-340 | 331-350 |
| Infected donor 1 | 0 | 0 | 0 | 0 | 0 | 0 | 0 | 0 | 0 | 0 | 0 | 0 | 0 | 0 | 0 | 0 | 0 |
| Infected donor 2 | 337 | 383 | 349 | 268 | 0 | 229 | 229 | 246 | 0 | 304 | 0 | 0 | 0 | 176 | 0 | 0 | 0 |
| Infected donor 3 | 0 | 0 | 0 | 0 | 0 | 0 | 0 | 0 | 0 | 0 | 0 | 0 | 0 | 0 | 0 | 0 | 0 |
| Infected donor 4 | 0 | 0 | 0 | 0 | 0 | 0 | 0 | 0 | 0 | 0 | 0 | 0 | 0 | 0 | 0 | 0 | 0 |
| Infected donor 5 | 0 | 0 | 0 | 0 | 0 | 0 | 0 | 0 | 0 | 0 | 0 | 0 | 0 | 0 | 0 | 0 | 0 |
| Infected donor 6 | 0 | 0 | 0 | 0 | 0 | 0 | 0 | 0 | 0 | 0 | 0 | 0 | 641 | 0 | 0 | 0 | 477 |
| Infected donor 7 | 1013 | 919 | 1251 | 915 | 911 | 921 | 0 | 0 | 1117 | 711 | 757 | 1063 | 759 | 649 | 799 | 805 | 685 |
| Infected donor 8 | 0 | 813 | 0 | 0 | 0 | 0 | 0 | 0 | 0 | 0 | 723 | 0 | 0 | 0 | 0 | 212 | 0 |
| Infected donor 9 | 0 | 263 | 0 | 0 | 0 | 0 | 0 | 0 | 0 | 0 | 0 | 0 | 0 | 0 | 0 | 0 | 0 |

| | **T cell response to anthrax PA domain I-IV epitopes, SFC/10⁶ cells** | | | | | | | | | | | | | | | | |
|---|---|---|---|---|---|---|---|---|---|---|---|---|---|---|---|---|---|
| **Human cohorts** | 341-360 | 351-370 | 361-380 | 371-390 | 381-400 | 391-410 | 401-420 | 411-430 | 421-440 | 431-450 | 441-460 | 451-470 | 461-480 | 471-490 | 481-500 | 491-510 | 501-520 |
| Infected donor 1 | 0 | 0 | 0 | 0 | 0 | 0 | 0 | 0 | 0 | 0 | 0 | 0 | 0 | 0 | 0 | 0 | 0 |
| Infected donor 2 | 0 | 263 | 325 | 304 | 0 | 263 | 215 | 268 | 323 | 299 | 0 | 232 | 179 | 222 | 0 | 325 | 289 |
| Infected donor 3 | 0 | 0 | 0 | 0 | 0 | 0 | 0 | 0 | 0 | 0 | 0 | 0 | 0 | 0 | 0 | 0 | 0 |
| Infected donor 4 | 0 | 0 | 0 | 0 | 0 | 0 | 0 | 0 | 0 | 0 | 0 | 0 | 0 | 0 | 0 | 0 | 0 |
| Infected donor 5 | 0 | 0 | 0 | 0 | 0 | 0 | 0 | 0 | 0 | 0 | 0 | 0 | 0 | 0 | 0 | 0 | 0 |
| Infected donor 6 | 0 | 0 | 0 | 0 | 0 | 0 | 0 | 0 | 0 | 599 | 801 | 0 | 0 | 0 | 577 | 0 | 0 |
| Infected donor 7 | 891 | 0 | 747 | 735 | 855 | 1199 | 0 | 0 | 859 | 1039 | 0 | 899 | 0 | 0 | 807 | 0 | 661 |
| Infected donor 8 | 0 | 0 | 0 | 0 | 0 | 0 | 0 | 0 | 0 | 0 | 0 | 0 | 0 | 0 | 0 | 0 | 0 |
| Infected donor 9 | 0 | 0 | 0 | 0 | 0 | 0 | 0 | 0 | 0 | 0 | 0 | 0 | 0 | 0 | 0 | 0 | 0 |

| | **T cell response to anthrax PA domain I-IV epitopes, SFC/10⁶ cells** | | | | | | | | | | | | | | | | |
|---|---|---|---|---|---|---|---|---|---|---|---|---|---|---|---|---|---|
| **Human cohorts** | 511-530 | 521-540 | 531-550 | 541-560 | 551-570 | 561-580 | 571-590 | 581-600 | 591-610 | 601-620 | 611-630 | 621-640 | 631-650 | 641-660 | 651-670 | 661-680 | 671-690 |
| Infected donor 1 | 0 | 0 | 0 | 0 | 0 | 0 | 0 | 0 | 0 | 0 | 0 | 0 | 0 | 0 | 0 | 0 | 0 |
| Infected donor 2 | 241 | 234 | 0 | 306 | 0 | 0 | 0 | 0 | 232 | 234 | 301 | 311 | 414 | 357 | 270 | 256 | 222 |
| Infected donor 3 | 0 | 0 | 0 | 0 | 0 | 0 | 396 | 0 | 0 | 0 | 0 | 0 | 0 | 0 | 0 | 0 | 0 |
| Infected donor 4 | 0 | 0 | 0 | 0 | 0 | 0 | 0 | 0 | 0 | 0 | 0 | 0 | 0 | 0 | 0 | 0 | 0 |
| Infected donor 5 | 1061 | 0 | 0 | 0 | 0 | 0 | 0 | 0 | 0 | 0 | 0 | 0 | 0 | 0 | 0 | 0 | 0 |
| Infected donor 6 | 0 | 0 | 603 | 0 | 0 | 0 | 495 | 779 | 0 | 0 | 0 | 0 | 539 | 0 | 0 | 0 | 545 |
| Infected donor 7 | 419 | 815 | 995 | 1391 | 881 | 1085 | 983 | 619 | 0 | 0 | 0 | 621 | 1043 | 0 | 893 | 705 | 993 |
| Infected donor 8 | 0 | 617 | 846 | 0 | 0 | 0 | 0 | 0 | 0 | 0 | 0 | 0 | 0 | 0 | 823 | 0 | 0 |
| Infected donor 9 | 0 | 0 | 0 | 0 | 596 | 0 | 0 | 0 | 0 | 0 | 0 | 0 | 0 | 0 | 474 | 0 | 0 |

| | **T cell response to anthrax PA domain I-IV epitopes, SFC/10$^6$ cells** | | |
|---|---|---|---|
| **Human cohorts** | 681-700 | 691-710 | 716-735 |
| Infected donor 1 | 0 | 0 | 0 |
| Infected donor 2 | 0 | 210 | 0 |
| Infected donor 3 | 532 | 0 | 0 |
| Infected donor 4 | 0 | 0 | 0 |
| Infected donor 5 | 0 | 0 | 0 |
| Infected donor 6 | 905 | 0 | 0 |
| Infected donor 7 | 929 | 1121 | 869 |
| Infected donor 8 | 0 | 0 | 0 |
| Infected donor 9 | 0 | 0 | 0 |

**Table S4. Differential susceptibility of HLA class II transgenic mice to anthrax infection**

| HLA | *B. anthracis* STI challenge dose (CFU) | Number of mice challenged | Number of challenge survivors | Mean time to death (days) | Bacterial load in spleens within observation period post-infection mean CFU/spleen (± SEM) | Bacterial load in spleens of survivors at day 20 mean CFU/spleen (± SEM) | Estimated $LD_{50}$ (CFU) |
|---|---|---|---|---|---|---|---|
| C57Bl6 | $10^5$ | 10 | 4 | 4.5 (±0.22) | $1.0x10^3$ ($±0.32x10^3$) (n=6) | 91 (±18) | $10^5$ |
| DQ6 | $10^5$ | 8 | 8 | - | - | 255 (±39) | $>10^5$ |
| DR4 | $10^5$ | 10 | 8 | 7 (±3) | $1.27x10^3$ (n=1) | 22 (±13) | $>10^5$ |
| DR15 | $10^5$ | 9 | 5 | 5.75 (±0.48) | $0.85x10^3$ ($±0.21\ x10^3$) (n=4) | 67 (±32) | $10^5$ |
| DQ8 | $10^6$ | 10 | 8 | 6 | $2.17x10^3$ ($±0.67x10^3$) (n=2) | 896 (±263) | $>10^6$ |
| DR1 | $10^6$ | 10 | 10 | - | - | 411 (±93) | $>10^6$ |

vaccines

MDPI

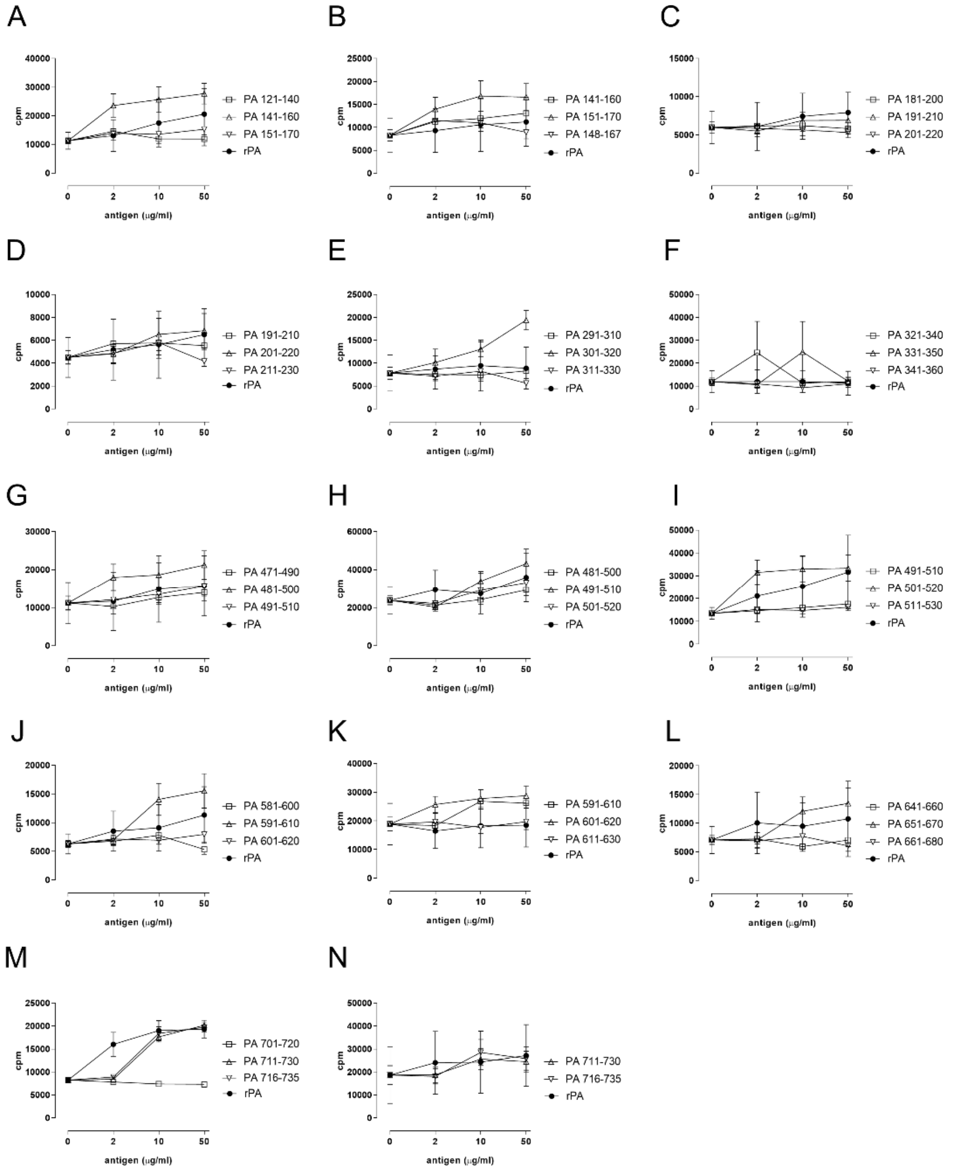


**Figure S1.** Fine specificity mapping of previously identified HLA-DQ8 restricted T cell epitopes.

HLA-DQ8 transgenics were immunised with the previously identified PA peptide in adjuvant. The proliferative responses of draining lymph node cells were measured in response to the indicated concentrations of whole PA protein, domains I-IV of the protein and the immunising and flanking peptides. The responses are shown as the stimulation index calculated as the mean CPM of triplicate wells in the presence of peptide divided by the mean CPM in the absence of antigen. Values twice the mean CPM in the absence of antigen were considered positive responses (n=3 for each data point).

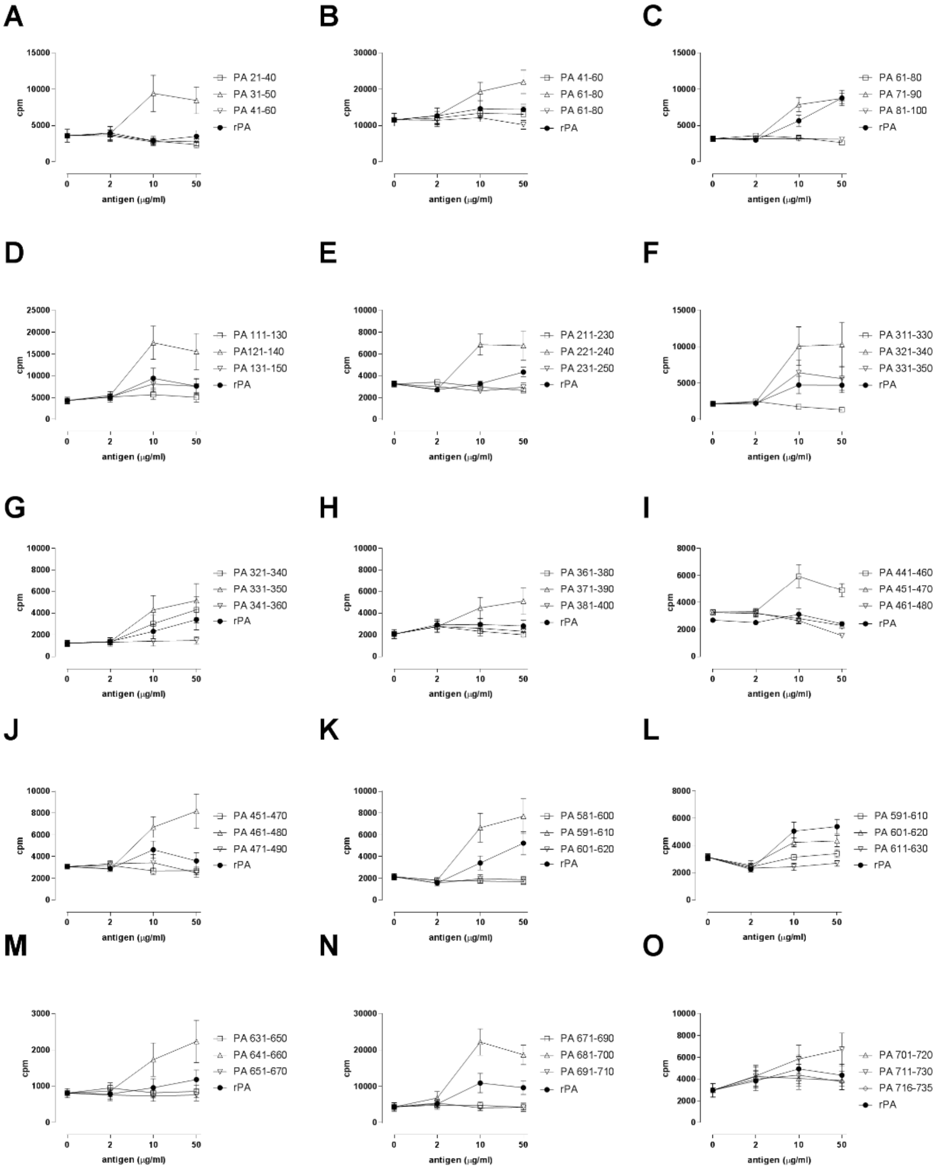


**Figure S2.** Fine specificity mapping of previously identified HLA-DR4 restricted T cell epitopes.

HLA-DR4 transgenics were immunised with the previously identified PA peptide in adjuvant. The proliferative responses of draining lymph node cells were measured in response to the indicated concentrations of whole PA protein, domains I-IV of the protein and the immunising and flanking peptides. The responses are shown as the stimulation index calculated as the mean CPM of triplicate wells in the presence of peptide divided by the mean CPM in the absence of antigen. Values twice the mean CPM in the absence of antigen were considered positive responses (n=3 for each data point).

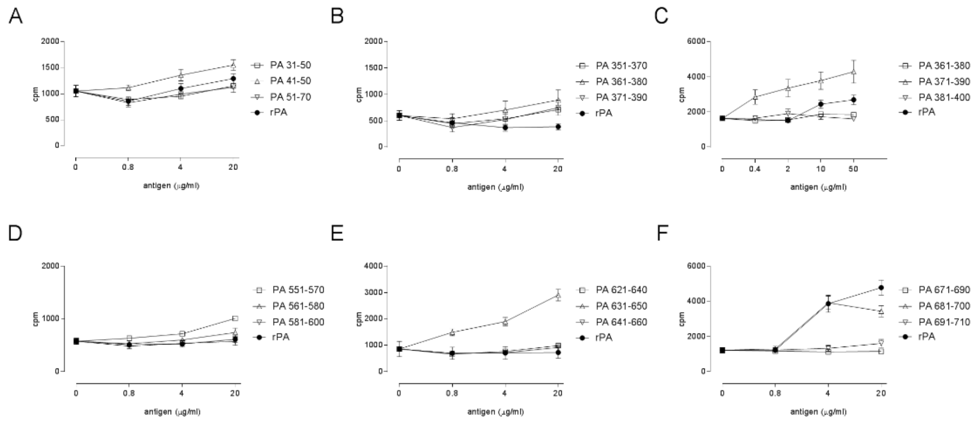


**Figure S3.** Fine specificity mapping of previously identified HLA-DR1 restricted T cell epitopes.

HLA-DR1 transgenics were immunised with the previously identified PA peptide in adjuvant. The proliferative responses of draining lymph node cells were measured in response to the indicated concentrations of whole PA protein, domains I-IV of the protein and the immunising and flanking peptides. The responses are shown as the stimulation index calculated as the mean CPM of triplicate wells in the presence of peptide divided by the mean CPM in the absence of antigen. Values twice the mean CPM in the absence of antigen were considered positive responses (n=3 for each data point).
